# True Courage

**Published:** 1878-08

**Authors:** 


True Courage—Is not so much marching up to the cannon's mouth in the hurry of battle, or mounting the scaffold for a principle, or enduring the surgeon's knife, as in living unknown and poor in a great city, striving hard, day by day, for daily bread, yet striving hopefully, resolutely, uncomplainingly and *rightfully.* Many a young heart *from the country of poor but pious parents*, comes every year to New York, and thus labors in hope of keeping dear ones at home, until life itself is worked out, and uncheered of any kindly world, unsustained by any helping hand, unaided by any pure philanthropist, unsought by any *man of God* whose mission is to seek out and *feed my lambs*, he goes down to the grave exclaiming, "*Thou, God,* ONLY *hast been my helper.*"

